# Assessment of mechanical, physical, and antimicrobial properties of tissue conditioners incorporated with plant extracts

**DOI:** 10.1007/s10266-025-01132-2

**Published:** 2025-06-13

**Authors:** Nevin Tas, Ferhan Egilmez, Sema Yiyit Dogan, Aysegul Hanife Mendi

**Affiliations:** 1 Kirikkale Provincial Health Directorate Kirikkale Oral and Dental Health Center, Kirikkale, Türkiye; 2 Department of Prosthodontics, Faculty of Dentistry, Gazi University, Bişkek Cd. 1.Sk. No:4 Emek, Ankara, Türkiye; 3 Technical Sciences Vocational School, Gazi University, Ankara, Türkiye; 4 Department of Basic Sciences, Division of Medical Microbiology, Faculty of Dentistry, Gazi University, Ankara, Türkiye

**Keywords:** Tissue conditioning, Stomatitis, Denture, Plant extracts

## Abstract

This study aimed to assess the antimicrobial effects of *Mentha piperita*, *Thymus vulgaris*, and *Salvia officinalis* extracts at varying concentrations (1%, 3%, 5%, and 10% by volume) against *S. mutans* and *C. albicans,* as well as to evaluate mechanical and physical properties, such as tensile bond strength, tear resistance, and surface roughness, after incorporating these extracts into acrylic-based tissue conditioners. The samples were prepared for the antimicrobial evaluation, comprising a negative control group (pure tissue conditioner), a positive control group (chlorhexidine digluconate and fluconazole), and test groups with plant extracts at different concentrations. The minimum inhibitory concentration (MIC) and agar disk diffusion methods were employed. Mechanical tests were conducted on samples containing the determined MIC. Antimicrobial test results were evaluated using Kruskal–Wallis and Mann–Whitney *U* tests, while mechanical properties were assessed by one-way ANOVA. A statistically significant difference in minimum inhibition zone diameters against *S. mutans* and *C. albicans* among the plant extracts at different concentrations was found (*p* < 0.001). However, no significant differences were observed between the groups in terms of tear resistance, tensile bond strength, and surface roughness (*p* = 0.198, *p* = 0.123, *p* = 0.094, respectively). 5% and 10% concentrations were found to be the most effective for positive control groups. 10% concentration of *Thymus vulgaris* demonstrated antimicrobial activity at a similar level to the 1% chlorhexidine digluconate for *S. mutans*. Additionally, incorporation of plant extracts at MIC level did not alter the tensile bond strength, tear resistance, or the surface roughness of the tissue conditioner.

**Supplementary Information:**

The online version contains supplementary material available at 10.1007/s10266-025-01132-2.

## Introduction

In patients using complete or removable partial dentures, movable tissues can be adversely affected by high stress concentrations during function. Significant damage may occur in the supporting tissues, leading to chronic pain, pathological changes, and bone loss during function. Due to the gradual changes in oral tissues, relining of complete or partial dentures is necessary to restore optimal adaptation of the denture base to the alveolar ridges and to ensure a more even distribution of occlusal forces on the supporting tissues [1].

Tissue conditioning materials distribute the energy generated by masticatory forces over a broader tissue area by absorbing loads that rigid denture bases cannot tolerate due to their inflexibility, thereby improving patient comfort and masticatory function [2]. Despite these advantages, tissue conditioners also have certain drawbacks. One of the most significant disadvantages is their lack of antimicrobial activity. The absence of antimicrobial efficacy facilitates the growth and colonization of various microorganisms, leading to increased denture-related complications [3].

Most microorganisms that colonize the oral cavity and lead to infections are not merely unicellular organisms; rather, they exist as intricate microbial communities [4]. Typically, these microorganisms are encapsulated within an exopolymeric matrix, which facilitates their adhesion to both biotic and abiotic surfaces, culminating in the formation of biofilms [4].

The hygiene of removable prostheses is intricately linked to the development of biofilms on the acrylic base and the supporting teeth. The plaque and biofilm layers encompass a diverse array of microorganisms, including bacteria and fungi [5]. This microbial biofilm represents a three-dimensional structure that has developed resistance to antimicrobial agents and immune cellular responses [4]. Notably, the environmental conditions beneath the prosthesis and the structural characteristics of the materials employed significantly contribute to this microbial proliferation and play a pivotal role in prosthesis-associated stomatitis [6]. Furthermore, removable prostheses that incorporate tissue conditioners become predisposing factors for microbial accumulation due to their interaction with the oral cavity’s microbial flora and associated inflammatory tissues [7]. Although it is possible to avoid denture stomatitis by changing denture hygiene habits and adequate oral care, it has been observed that traditional mechanical and chemical denture cleaning methods affect the properties of the lining materials [8]. Consequently, the use of secondary metabolites derived from medicinal and aromatic plants has recently emerged as a noteworthy approach in the development of alternative cleaning methods.

The surfaces within the oral cavity exhibit pronounced colonization, particularly by *S. mutans* and other streptococci, which are primary contributors to caries formation. Additionally, it has been observed that the presence of streptococci enhances the adherence of fungi such as *C. albicans* to prosthetic surfaces [9]. Within the oral mucosa, *C. albicans* is predominant, accounting for approximately 75%, followed by *C. tropicalis* at 8%, with lower prevalence rates of *C. krusei* and *C. glabrata* [10, 11]. The excessive presence of *C. albicans* significantly escalates the demand for local or systemic antifungal agents, given its critical role in infection pathogenesis [11]. However, Candida infections have evolved into a significant health concern, attributed to the increasing resistance of these fungi to existing therapeutic agents and the high rates of recurrence.

In the management of oral fungal infections, topical treatment constitutes the initial therapeutic approach. Nevertheless, in instances of poor patient compliance, the efficacy of topical application diminishes, prompting the incorporation of antifungal agents into tissue conditioners as an alternative strategy. However, the indiscriminate use of antifungal agents has been linked to the emergence of resistant Candida strains, thereby highlighting the potential risks associated with this approach [12].

Given these challenges, along with the rising issues of antibiotic resistance and toxicity, there has been a concerted effort to develop alternative agents derived from natural sources [13]. Moreover, since 1977, the World Health Organization (WHO) has actively promoted and developed programs aimed at enhancing traditional medicine practices grounded in the utilization of medicinal plants [14]. Consequently, there is an emphasis on leveraging a diverse range of plant species with various therapeutic properties. Thus, the potential of natural plant extracts and oils to inhibit the growth and proliferation of microorganisms involved in biofilm formation has emerged as a long-standing area of research within dental literature.

The primary objective of this study is to evaluate the antimicrobial and antifungal efficacy of tissue conditioners formulated with three distinct natural plant extracts against *S. mutans* and *C. albicans*. Following this, it is aimed to evaluate the physical and mechanical properties of the tissue conditioner material after the incorporation of these extracts, using the effective dosages determined.

This investigation has three hypotheses: (1) There is no significant difference in the antimicrobial and antifungal efficacy among the tested tissue conditioner materials following the addition of plant extracts. (2) There is no significant variation in the antimicrobial and antifungal efficacy across different concentrations of the plant extracts. (3) The addition of plant extracts at minimum inhibitory concentrations (MICs) to tissue conditioners influences the mechanical and physical properties of the tissue conditioning material.

## Materials and methods

The product names, compositions, lot numbers, manufacturers, and material contents of the materials used in the study are detailed in Supplementary file.

### Preparation of culture media, microorganisms, and plant extracts

The media utilized in this study were dissolved in 1 liter of distilled water and sterilized in an autoclave (MaXterile 47, Gangwon-do, Korea) at 121 °C for 15 minutes. The microorganisms used, specifically *C. albicans* ATCC#10231 and *S. mutans* ATCC#25175, were cultivated by inoculating them into liquid media: Sabouraud Dextrose Broth (SDB) (1.08339; Merck, Darmstadt, Germany) and Brain Heart Infusion Broth (BHI) (1400; Condalab, Spain), respectively, at a 2% concentration and incubated at 37 °C for 24 hours. After cultivating the microorganisms, they were activated twice and centrifuged at 5000 rpm for 10 minutes. The supernatant was discarded, and phosphate-buffered saline (PBS) was added to the pellet, which was washed twice. Following the washing process, the pellet was reconstituted in PBS to achieve a McFarland 0.5 density for antimicrobial studies.

In this study, endemic plants such as *Mentha piperita*, *Salvia officinalis*, and *Thymus vulgaris* were collected from a local market in Ankara, Türkiye. A 10% dimethyl sulfoxide (DMSO) (Merck, Darmstadt, Germany) solution was employed for adjusting the concentrations of the extracts, while methanol (Merck, Darmstadt, Germany) served as the solvent for obtaining the plant extracts. A hot air oven at 60 °C was used to dry the plant materials for 72 h. Then, they were grounded to coarse powders. Each pulverized herbs were weighed at 2 g each, then placed in separate Erlenmeyer flasks containing 20 mL of methanol in different flasks to obtain crude methanolic extracts and kept at room temperature for 12 h. 10 g of each remaining residue powders in the bottles was mixed with 100 mL of sterile, deionized water and incubated in the dark at 20 °C for 3 days. The mixture was gently agitated by hand for 30 s. daily. Following incubation, the extract was sterilized through a 0.2 µm membrane filters. This preparation was defined as the full-strength extract (100%). Lower concentrations for experimental use were prepared through serial dilution of the full-strength extract.

### Preparation of acrylic-based tissue conditioner samples for antimicrobial activity

A perforated polytetrafluoroethylene mold was utilized to obtain 340 disk-shaped acrylic-based tissue conditioner (GC Tissue Conditioner, GC Dental Products Leuven, Belgium) samples (*ø* = 10 mm, *h* = 1 mm) to evaluate antimicrobial properties (*n* = 10/subgroup).

The sample size was calculated with the G*Power software (version 3.1.9.6) based on a previous study [15] to estimate the power of 0.85 at *α*  =  0.05. Accordingly, the minimum number of samples per subgroup was determined as 9. Considering that the possible sample loss during the experiments, the number n was taken as 10 for the antimicrobial, antifungal experiments and mechanical tests [16].

**Fig. 1:**
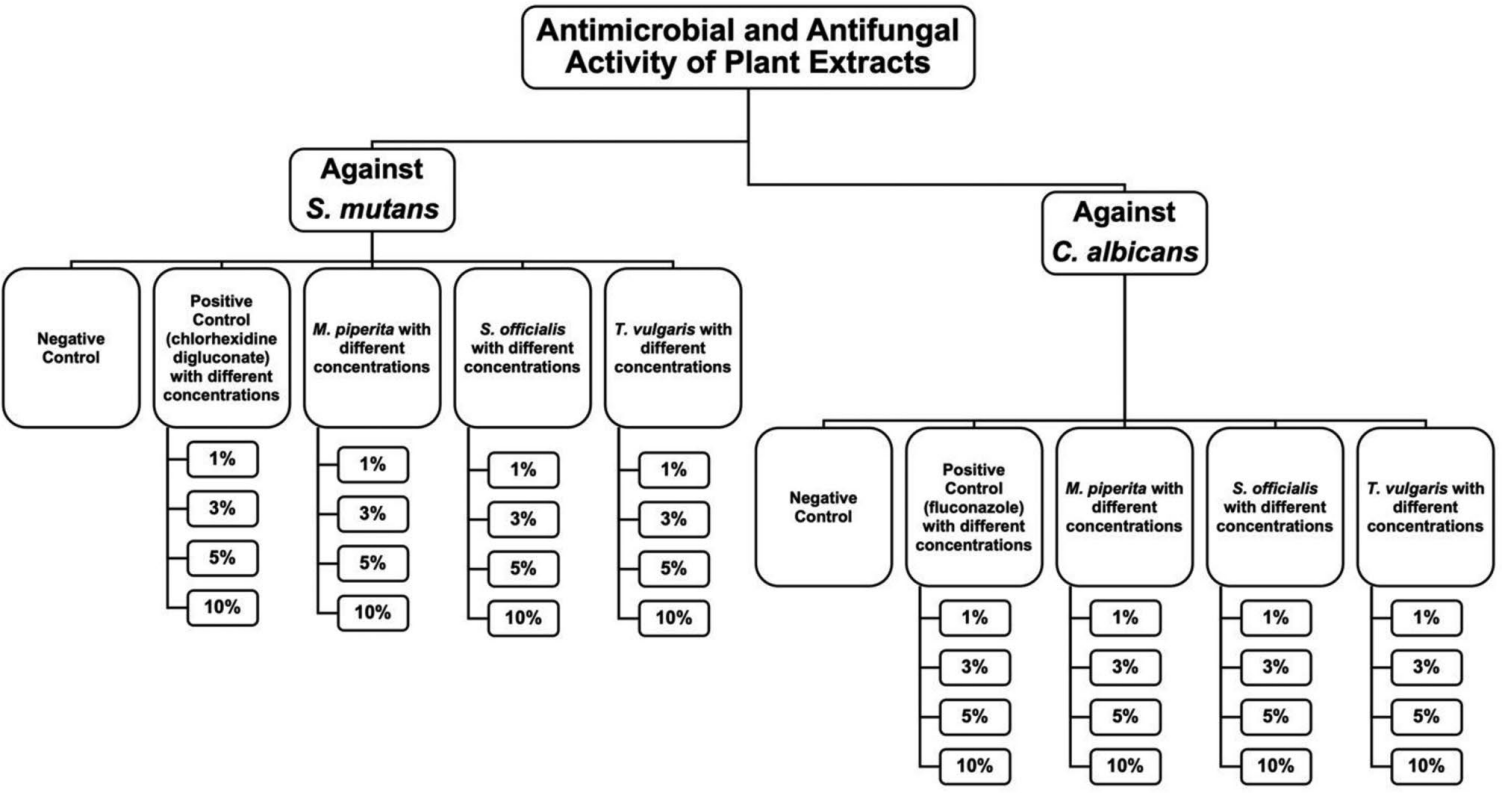
Schematic study design

**Fig. 2:**
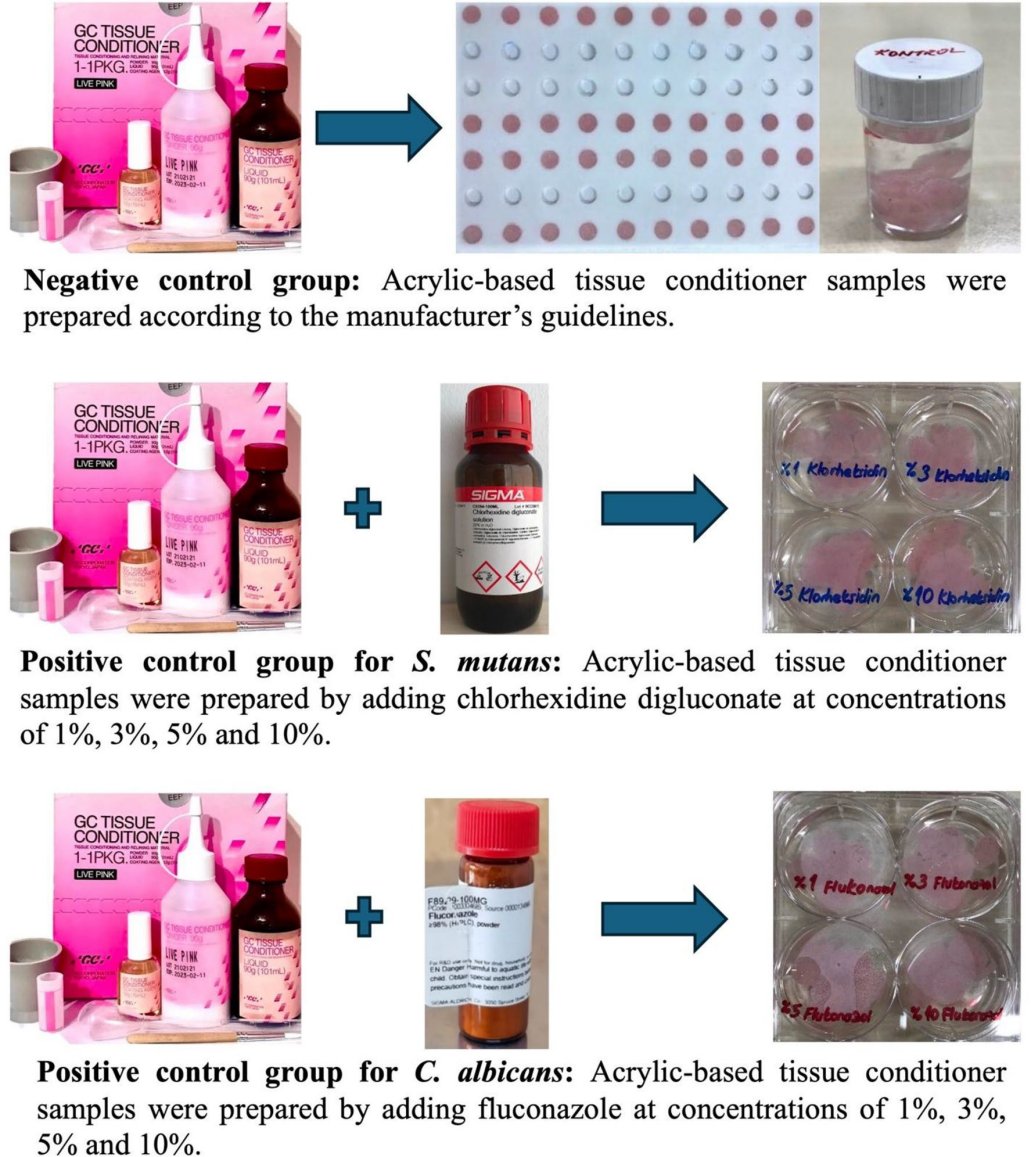
Preparation of negative and positive control samples

Schematic study design is given in Fig. 1. The negative control comprised acrylic-based tissue conditioner samples contain no plant extracts or antimicrobial agents, prepared according to the manufacturer’s guidelines (Fig. 2). For this purpose, 2.4 g powder and 2 mL liquid were dispensed into a rubber cup and mixed for 60 sec. until the mixture becomes creamy. Then, this mixture was placed into perforated polytetrafluoroethylene mold and waited for 5 min.

For the positive control group, disk samples containing chlorhexidine digluconate solution (Sigma-Aldrich, St. Louis, USA) and fluconazole powders (Sigma-Aldrich Massachusetts, USA) were incorporated by mixing with the powder and liquid of the acrylic-based tissue conditioner in concentrations of 1%, 3%, 5%, and 10% (v/v), following the manufacturer’s instructions (Fig. 2).

**Fig. 3:**
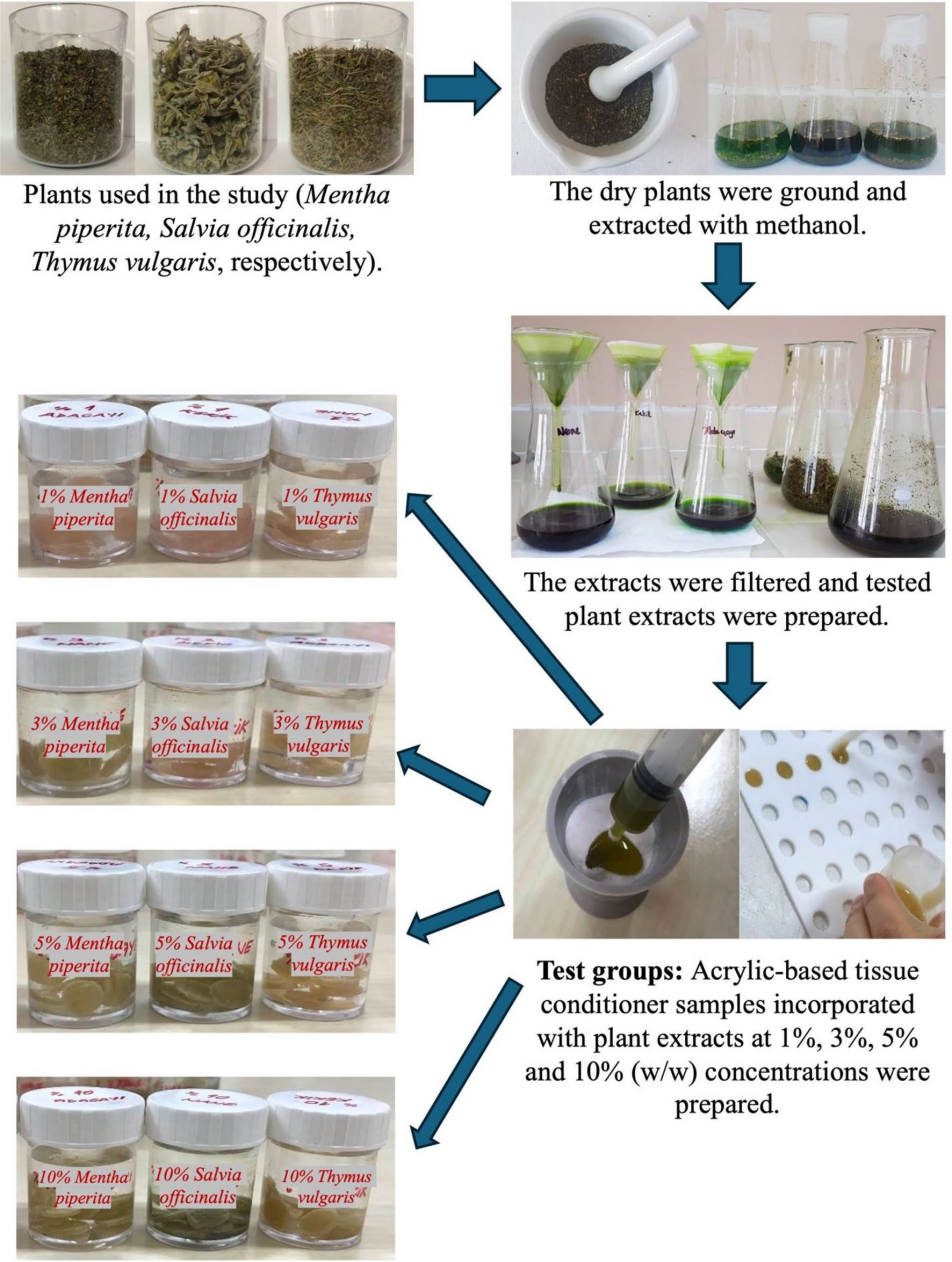
Preparation of samples of test groups

For the test groups, plant extracts of *Mentha piperita, Salvia officinalis,* and *Thymus vulgaris* were measured volumetrically with a micropipette to ensure an extract concentration of 1%, 3%, 5%, and 10% by volume according to the recommended powder/liquid ratio by the manufacturer, then mixed with the liquid component of the tissue conditioner. Subsequently, the liquid incorporated with plant extracts was mixed with the powder of acrylic-based tissue conditioner material as per the manufacturer’s guidelines (Fig. 3). The plant extracts and the powder of tissue conditioner material were pre-volumed

All samples from the negative control, positive control, and test groups were removed from the mold and allowed to stand in distilled water for 1-week to allow for monomer leaching. They were then disinfected with 70% alcohol for 1 min. and rinsed with distilled water. Thereafter, the negative control, positive control, and test samples were placed on petri dishes inoculated with microorganisms and incubated at 37 °C for 7 days.

### Determination of antimicrobial activity

#### Minimum Inhibitory Concentration (MIC) determination of plant extracts

The microdilution method was used to determine the MIC values of positive control groups and plant extracts. For this purpose, 100 μL of BHI broth medium was poured into 96 well bottom microplate wells. Then, positive control solutions and various concentrations of the plant extracts (1%, 3%, 5%, and 10%) were inoculated separately in each row of the microplate. Then, the media containing the positive control solutions and plant extracts were inoculated with the suspension of *S. mutans* and *C. albicans*, adjusted to a McFarland 0.5 density, and incubated at 37 °C for 24 hours. After incubation, the concentration that exhibited no turbidity was considered the MIC [17, 18].

#### Determination of Minimum Inhibition Zone (MIZ) diameters using the agar disk diffusion method

The antimicrobial and antifungal activity of tissue conditioners incorporated with plant extracts released from disks on *S. mutans* and *C. albicans* was assessed by using the agar disk diffusion assay. For this test, *S. mutans* and *C. albicans* were prepared with 0.5 McFarland’s microbial suspension (1.5 × 10^8^ CFU/mL) and inoculated onto BHI and SDB agar plates, respectively.

Wells of 6 mm were created in the agar plates using a sterile punch. All samples including negative control, positive control, and test groups were placed into wells and incubated at 37 °C for 24 h. After incubation, the inhibition zones surrounding the samples were measured by using a digital veneer caliper to determine efficacy [17, 18]. The mean inhibition zones were calculated. All experiments were conducted in triplicate at 24 hours and 7 days post-incubation.

### Preparation and testing of samples for mechanical and physical tests

After the determination of MIC of the tested plant extracts mechanical tests were performed with freshly prepared samples. A priori power analysis based on a previous study [19] revealed 7 tissue conditioner test samples incorporated with the plant extracts at their MICs per group were deemed sufficient (*f* = 0.5, 1 – *α* = 0.05, 1 − *β* = 0.90) for the mechanical and physical tests. Considering this result, a total of 120 samples (*n* = 10) were prepared [16].

Three different tests were utilized for this purpose. For the mechanical and physical tests, tissue conditioner test samples were incorporated with the plant extracts at their MICs for *C. albicans.*

#### Tear resistance test

For tear resistance measurements, samples without plant extracts (control group) and test samples were prepared in bar-shape (50 × 10 × 1 mm^3^) using aluminum molds, in accordance with ISO 34-1 standards [20]. For the tear resistance tests, both the control groups and the test groups containing plant extracts at MICs were cut into “trouser” shapes using a #15 scalpel (25 mm long). The sections resembling the legs of the trouser were arranged vertically in opposing directions. A universal testing machine (Lloyd-LRX; Lloyd Instruments, West Sussex, UK) was utilized to record the maximum load in Newtons for each sample.

#### Tensile bond strength test

Tensile bond strength between heat-polymerized acrylic resin denture base material (Lcad Dent Hot Curing Denture Base Material, Hamle Tıbbi Cihazlar Malz. Tic. Ltd. Şti., İzmir, Turkiye) and tissue conditioner incorporated with plant extract at MIC samples for *C. albicans* was measured. For this measurement, dumbbell-shaped wax samples (75 × 25 × 2 mm^3^) were prepared in aluminum molds following ISO 37 standards [21]. The prepared wax samples were measured in two separate pieces to ensure a 3 mm gap between two plates and placed in the plaster in the flask for conventional compression molding technique using a long polymerization cycle.

Prepared heat-polymerized acrylic resin plates were finished and polished. Subsequently, the surfaces of each acrylic resin plate to be bonded were standardized by abrading with 100-grit sandpaper. The polymerized acrylic resin samples were placed back into the aluminum mold. The negative control and test group tissue conditioner samples were prepared and positioned in the 3 mm gap. Tensile bond strength measurements were conducted at a speed of 0.5 mm/min using a universal testing machine (Lloyd-LRX; Lloyd Instruments, West Sussex, UK), with the values obtained in Newtons divided by the surface area of the samples to calculate the tensile bond strength in MPa.

#### Surface roughness test

The negative control and test samples (25 mm in diameter and 15 mm in thickness) were prepared by using a 4-piece aluminum mold. Thereafter, all the samples were left in distilled water for one week.

Surface roughness (Ra, µm) was measured using a portable profilometer (Perthometer M2, Mahr GmbH, Göttingen, Germany). Before measuring the test samples, the device was calibrated using a sample provided by the manufacturer. All samples were placed on a stable, vibration-free table, ensuring the profilometer’s recording tip was perpendicular to the sample surface. Surface roughness measurements were recorded as the recording tip traversed a 0.8 mm length across the sample. A total of three recordings were taken from each sample over a total recording length of 2.4 mm to determine average Ra values. The device was recalibrated before each test group measurement.

#### Topographic analysis using scanning electron microscopy

Topographic analyses of the tested samples were performed using a scanning electron microscope (JEOL-JSM 6060 LV, Tokyo, Japan). After gold-palladium coating, two samples from each test group were examined at 15 kV and 10 mm working distance, using magnifications of 2000 and 5000, focusing on their surface and structural morphologies.

### Statistical method

Statistical evaluations were conducted using the SPSS 15.0 software package (IBM Corp., Chicago, Illinois, USA). The normality of continuous numerical variables was assessed using the Kolmogorov–Smirnov test, while homogeneity of variances was investigated with Levene’s test. For continuous numerical variables that did not conform to a normal distribution, the significance of differences among test groups was examined using the Kruskal–Wallis test. When there were two independent groups, the Mann–Whitney *U* non-parametric comparison test was used to assess significance between groups. The statistical analysis of normally distributed data was conducted using one-way ANOVA. A *p*-value of <0.05 was considered statistically significant. To control for potential Type I errors in all multiple comparisons, Bonferroni correction was applied.

## Results

### Antimicrobial activity results

*Minimum inhibitory concentration (MIC) results of plant extracts*

The MIC values of tested groups for *S. mutans* and *C. albicans* are given in Table 1.

**Table 1:** Minimum inhibitory concentrations (MICs) of plant extracts

	MIC (mg/mL)	
	*S. mutans*	*C. albicans*
Chlorhexidine Digluconate	<0.2	–
Fluconazole	–	<0.2
*Mentha Piperita*	0.4	3.13
*Salvia Officinalis*	1.56	12.5
*Thymus Vulgaris*	0.4	1.56

*Minimum inhibition zones (MIZ) results*

#### Antibacterial activity results

The Kolmogorov–Smirnov test results indicated that inhibition zone values of the independent variables for the *S. mutans* microorganism did not conform to a normal distribution (*p* < 0.05). Notably, samples from the negative control group, which did not incorporate plant extracts, failed to yield any inhibition zone against *S. mutans* (MIZ = 0).

The MIZ values against *S. mutans* revealed a statistically significant difference among the various plant extracts (*H* = 93.024, *p* < 0.001). Furthermore, a significant disparity was also observed among the zone diameters generated by the different concentrations of plant extracts against *S. mutans* (*H* = 36.612, *p* < 0.001).

Mann–Whitney *U* non-parametric intergroup comparison test revealed that disregarding the concentrations of the plant extracts, the overall ranking of plant extract types in terms of the MIZ against *S. mutans* is delineated as follows:

Negative control < *Mentha piperita* < *Salvia officinalis* ≤ *Thymus vulgaris* < chlorhexidine digluconate.

As a result of the statistical evaluation, the ranking of the MIZs determined according to the concentrations of plant extracts against *S. mutans* is as follows:

1% < 3% < 5% ≤ 10%.

**Fig. 4:**
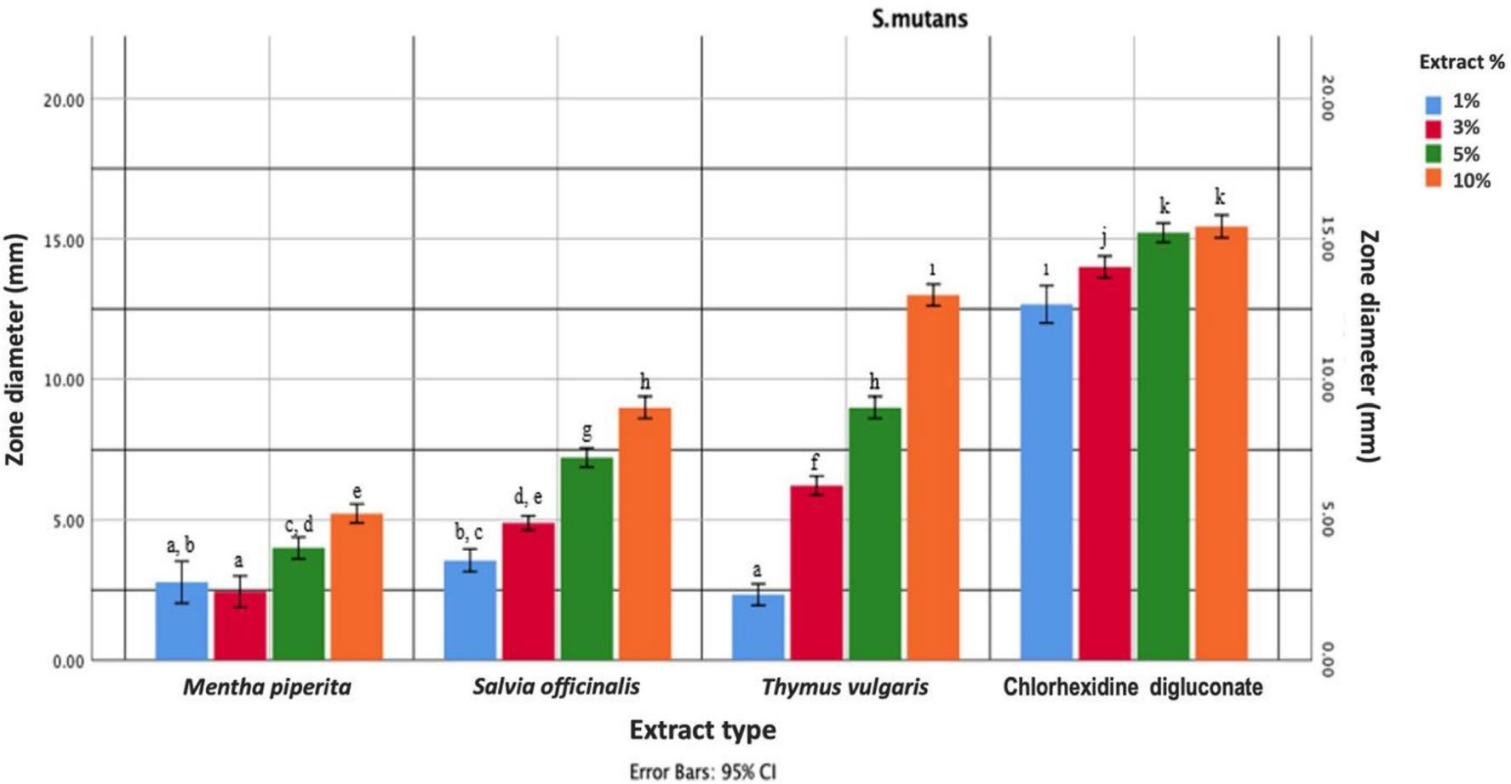
The minimum inhibition zones (MIZs) created by various plant extracts and chlorhexidine digluconate against *S. mutans*. Different lowercase letters indicate significant differences. Error bars represent the 95% confidence intervals of mean difference

The interaction between independent variables in terms of the MIZs induced by plant extracts and various concentrations against *S. mutans* was found statistically significant different (*F* = 630.731; *p* < 0.001). Additionally, it was observed that the 3%, 5%, and 10% concentrations of chlorhexidine digluconate exhibited statistically greater efficacy than all tested plant extracts and their concentrations (*p* < 0.0125). Furthermore, this efficacy was followed by the 1% concentration of chlorhexidine digluconate and the 10% concentration of *Thymus vulgaris* (*p* < 0.0125) (Fig. 4).

#### Antifungal activity results

In accordance with the Kolmogorov–Smirnov test results, it was determined that the inhibition zone values of independent variables for *C. albicans* also did not exhibit a normal distribution (*p* < 0.05). The negative control group samples, without plant extracts, did not produce an inhibition zone on *C. albicans* (MIZ = 0).

According to the Kruskal–Wallis test results, there was a statistically significant difference in MIZ diameters against *C. albicans* among the plant extracts (*H* = 85.817; *p* < 0.001). It was observed that fluconazole formed a larger inhibition zone compared to the plant extracts prepared at MIC.

The non-parametric Mann–Whitney *U* test for pairwise comparisons revealed that the plant extract types were ranked according to the MIZs they formed against C*. albicans*, regardless of concentration, was as follows:

Negative control < *Salvia officinalis* ≤ *Mentha piperita* ≤ *Thymus vulgaris* < fluconazole.

The Kruskal–Wallis test results showed that the extract the capacity to form inhibition zone among extract concentrations varied at different concentrations (*H* = 31.946; *p* < 0.001).

In addition, the ranking of extract concentrations in terms of MIZ against *C. albicans* is as follows:

1% ≤ 3% < 5% ≤ 10%.

**Fig. 5:**
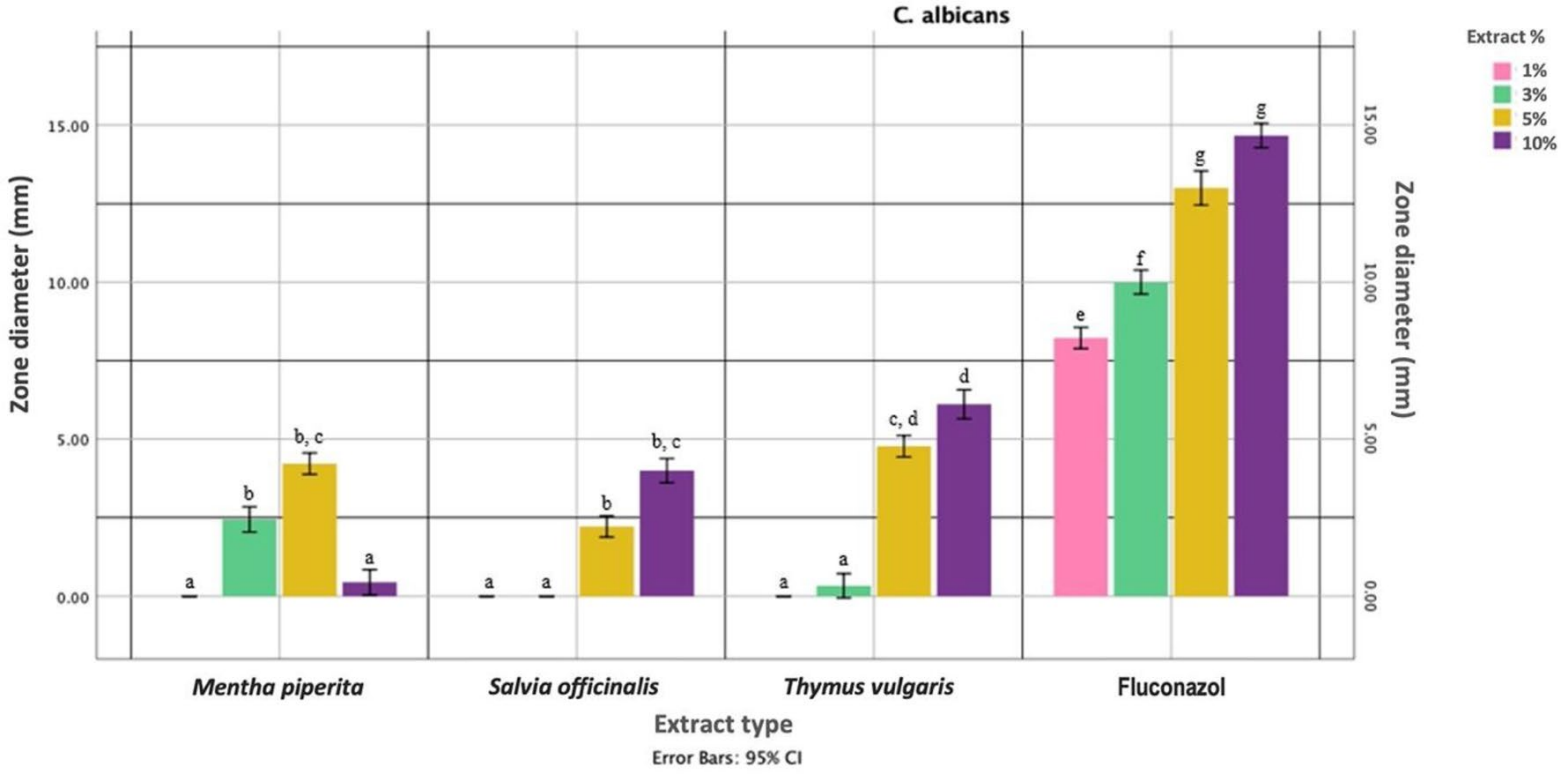
Minimum inhibition zones (MIZs) of different plant extracts and fluconazole against *C. albicans*. Different lowercase letters indicate significant differences. Error bars represent the 95% confidence intervals of mean difference

A statistically significant difference was found as a result of analyzing the interaction between plant extract types and concentrations for MIZ formation against *C. albicans* (*F* = 262.623; *p* < 0.001). Fluconazole at all concentrations formed a significantly larger MIZ than all plant extract concentrations (*p* < 0.0125) (Fig. 5).

### Physical and mechanical properties results

#### Tear resistance test results

**Fig. 6:**
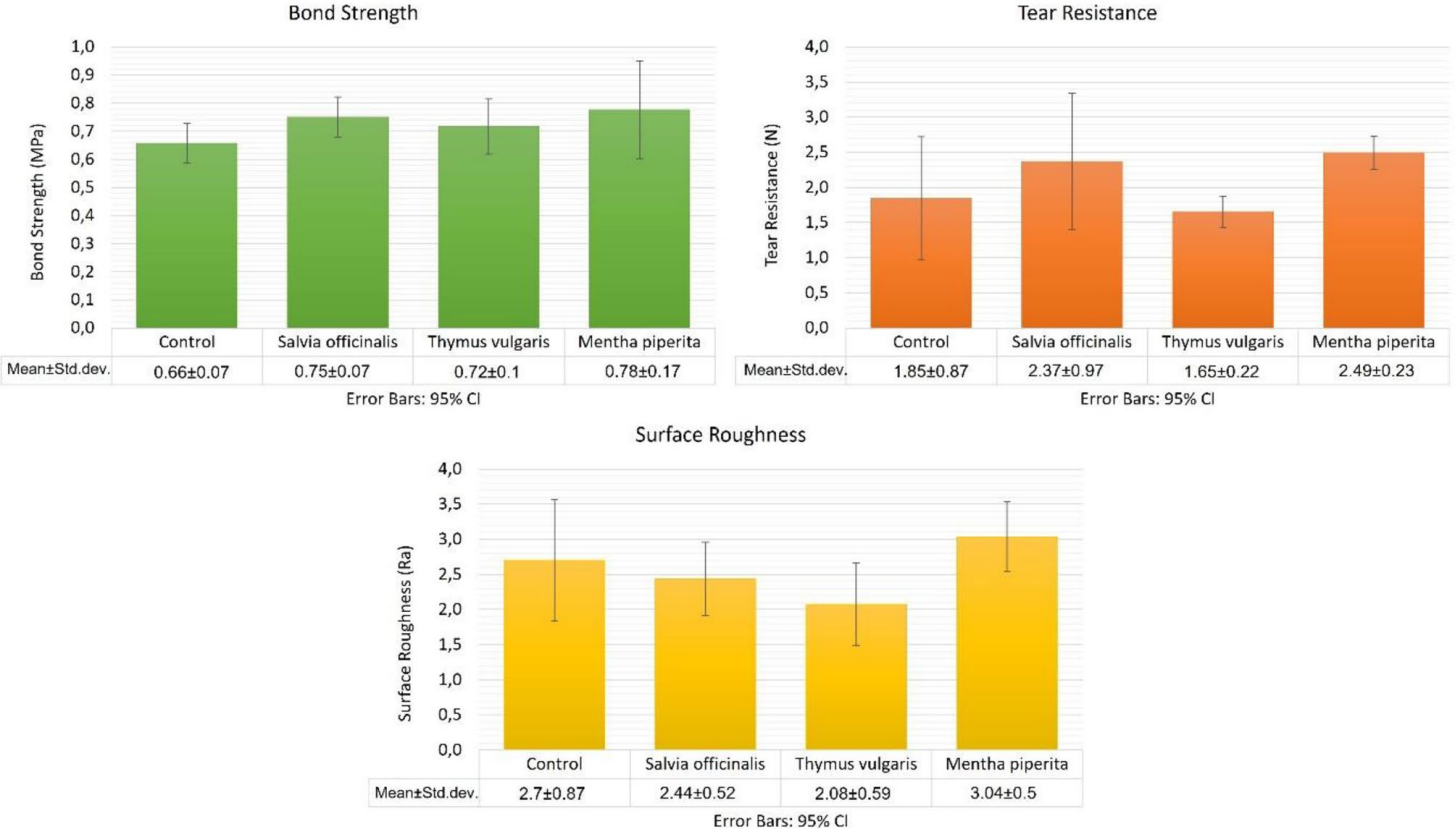
Results of bond strength, tear resistance, and surface roughness tests for tissue conditioners containing plant extracts at minimum inhibitory concentration (MIC) and the control group. Error bars represent the 95% confidence intervals of mean difference

The tear resistance test results showed no statistically significant difference between the negative control group and test groups (tissue conditioner samples incorporated with plant extracts at MICs) (*F* = 2.172; *p* = 0.123). The results are depicted in Fig. 6.

#### Tensile bond strength test results

Tensile bond strength values between heat-polymerized acrylic resin denture base material and tissue conditioner samples incorporated with plant extract at MIC level exhibited no statistically significant difference (*F* = 1.659; *p* = 0.198). The graphical representation of these results is shown in Fig. 6.

#### Surface roughness results

The results of the surface roughness test indicated no statistically significant difference between the control group and the tissue conditioners incorporating plant extracts at MIC (*F* = 2.446; *p* = 0.094). The statistical results are illustrated in Fig. 6.

### Scanning electron microscope (SEM) results

**Fig. 7:**
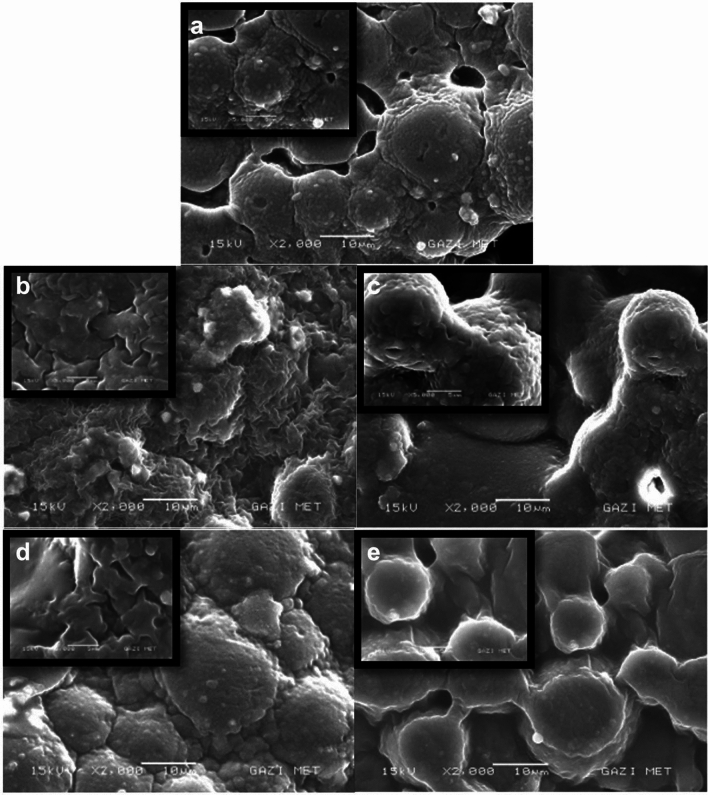
Representative SEM images of tissue conditioning samples tested in this study. (15 kV, x2000). **a** control group, **b** *Thymus vulgaris* extract incorporated at 3% concentration **c** *Thymus vulgaris*––10% concentration, **d** *Mentha piperita* extract incorporated at 3% concentration, **e** *Mentha piperita*––10% concentration

The images obtained from the SEM were exhibited that in tissue conditioning samples containing *Thymus vulgaris* extract, the surface roughness decreased as the concentration ratio increased. Additionally, the images exhibited more similar images to the control group (Fig. 7a–c).

When the tissue conditioner samples containing *Mentha piperita* extract were compared with the control group it was seen that more similar surface characteristics were obtained. In addition, it was observed that increasing the plant extract concentration decreased the roughness (Fig. 7d, e).

**Fig. 8:**
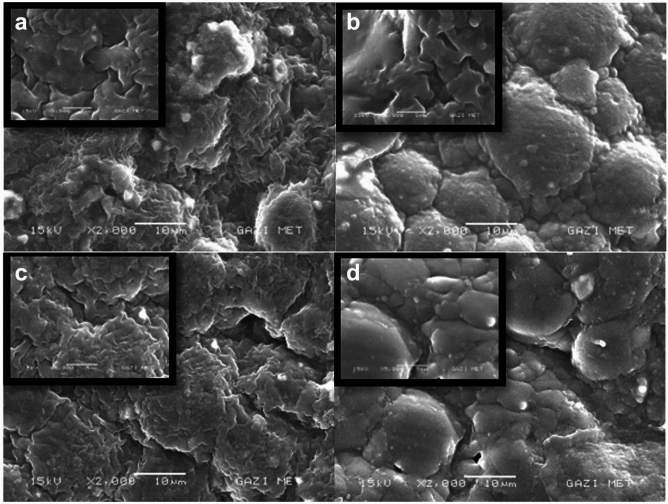
Representative SEM images of tissue conditioner samples containing 3% and 5% plant extracts (15 kV, x2000): **a** *Thymus vulgaris*––3% concentration, **b** *Mentha piperita*––3% concentration, **c** *Salvia officinalis*––5% concentration, **d** *Thymus vulgaris*––5% concentration

When tissue conditioner samples containing 3% concentration were compared with the control group, it was observed that tissue conditioner samples containing *3% Mentha piperita* extract exhibited a more similar morphology to the pure tissue conditioner (control group) (Fig. 8a, b). When 5% *Salvia officinalis* and 5% *Thymus vulgaris* concentrations were compared, it was seen that the appearance of 5% *Thymus vulgaris* samples was more closely resembled to the control group compared to 5% *Salvia officinalis* (Fig. 8c, d). In brief, no difference was observed between different groups in morphology. In brief, no difference was observed between different groups in morphology.

## Discussion

Anatolia and Mediterranean regions, including Türkiye, France, Portugal, Spain, Italy, and Greece, have diverse topographies, and their climates have fostered a unique botanical tradition, with numerous plant species exhibiting medicinal properties [22]. Of particular interest are Anatolian medicinal plants, such as *Echinacea purpurea L Moench, Achillea filipendulina Lam., Salvia officinalis, Mentha piperita L., Lavandula angustifolia, and Thymus vulgaris*, known for their bioactive compounds with antimicrobial, antioxidant, anticancer and antifungal potential [23]. Therefore, this study aimed to test the antibacterial and antifungal properties of three selected Anatolian medicinal plants those targeting the oral pathogens such as *S. mutans* and *C albicans*.

For this purpose, we evaluated the effects of incorporating three different plant extracts and two antimicrobial agents into an acrylic-based tissue conditioner and analyzed their mechanical, physical, and antimicrobial properties. The first hypothesis that there would be no difference in the antimicrobial and antifungal effectiveness of the conditioned materials post-plant extract addition was rejected due to statistically significant differences observed in the antimicrobial activities of the plant extracts.

The second hypothesis that there would be no difference in efficacy between different concentrations of plant extracts was also rejected due to the statistical significance of concentration-dependent changes in antimicrobial activity. Our third hypothesis suggested that plant extracts added at MIC levels would affect the mechanical and physical properties of the tissue conditioner. However, this hypothesis was rejected as there were no significant differences in tensile bond strength, tear resistance, or surface roughness between experimental and control samples when extracts were added at MIC levels.

Various methods are used to evaluate the antimicrobial efficacy of agents added to dental materials, including agar or disk diffusion, MIC, and broth dilution methods [24]. However, these methods primarily yield short-term results on bacterial viability while providing a preliminary indication of potential antimicrobial efficacy [25].

The MIC values for the antimicrobial effects of plant extracts against *S. mutans* in our study were 1.56 mg/mL for *Salvia officinalis*, and 0.4 mg/mL for both *Mentha piperita* and *Thymus vulgaris*. The MIC value for the antimicrobial agent chlorhexidine digluconate used as a positive control was found to be <0.2 mg/mL.

In a previous study, it was demonstrated that the MIC of menthol, the primary antimicrobial compound in *Mentha piperita*, against *S. mutans* is approximately 15.6 µg/mL at a concentration of 200 µg/mL [26]. Another study, which examined 14 essential oils, including *Mentha piperita*, *Thymus vulgaris*, and *Salvia officinalis*, reported MICs of 16 µg/mL for *Salvia officinalis* and *Thymus vulgaris*, 4 µg/mL for *Mentha piperita*, and 2 µg/mL for chlorhexidine against *S. mutans* [27].

The results of the current study are in line with the research of Mirpour *et al.* [28], who evaluated the antimicrobial efficacy of *Thymus vulgaris* against *S. mutans* and *S. salivarius* and reported an MIC of 1.55 mg/mL for *Thymus vulgaris*. Gylan *et al.* [29] observed an MIC of 0.156 mg/mL for *Salvia officinalis* against *S. mutans*, while they observed an MIC of 0.02 mg/mL for chlorhexidine, highlighting the variability in MICs observed between different extracts.

There are numerical differences between the effective values due to the MIC measurement being made at different concentrations in dental literature. Our results demonstrated effective antimicrobial activity of all tested plant extracts against *S. mutans* at MIC levels. However, chlorhexidine digluconate showed a higher level of efficacy than the plant extracts, consistent with other findings [30]. According to the available study data, chlorhexidine shows antibacterial activity at physiological pH [30]. It has been reported that the bactericidal activity of chlorhexidine is due to the release of a cation produced when the chlorhexidine salt decomposes and causes cell death in microorganisms [31].

Regarding antifungal properties, MIC values against *C. albicans* were 12.5 mg/mL for *Salvia officinalis*, 3.13 mg/mL for *Mentha piperita*, and 1.56 mg/mL for *Thymus vulgaris*, with the positive control antifungal agent fluconazole exhibiting an MIC of <0.2 mg/mL. Our findings align with the antifungal efficacy of *Mentha piperita* essential oil against *C. albicans* in previous studies, though fluconazole was more potent [32].

However, in some studies where fluconazole was found to be less effective [33, 34], the variability between the antibacterial and antifungal effects determined by considering the MIC values of the tested plants may be due to different environmental or experimental factors [35]. The extraction procedures, test methods, and strains of the tested microorganisms are experimental factors affecting the results of antifungal effects [36]. It has also been reported that environmental factors such as climate, season, geological condition, and harvest period can significantly affect the biological properties of plants such as their active chemical composition [35]. Therefore, it is thought that the reason for the differences in the study results may also be due to the differences in the main components of essential oils and plant extracts depending on geographical conditions, climate, and the effect of sunlight [37].

When the results of our study were examined, regardless of the plant extract concentrations, it was found that *Mentha piperita* extract had the least activity in terms of the MIZ formed by the plant extract types against *S. mutans*, and the highest antimicrobial activity was exhibited by the test group containing chlorhexidine digluconate.

In the study by Songsang et al. [15] investigating the antimicrobial properties of tissue conditioners (GC, Viscogel and Coe comfort) added with various concentrations of *Litsea cubeba* essential oil against *S. mutans* and *C. albicans*, 2% chlorhexidine was used as the positive control group. As a result of the study, no antimicrobial activity was observed in the negative control group, while it was observed that chlorhexidine formed significantly more zones than the other test groups containing essential oils [15].

Raghavan *et al.* [38] used 0.2% chlorhexidine as a positive control group in their in vitro studies investigating the effects of *Mentha piperita* leaf extracts against oral pathogens (*S. mutans, A. actinomycetemcomitans,* and *C. albicans*). The researchers found that chlorhexidine created more MIZ against both *S. mutans* and *C. albicans* [38]. Our study results are parallel to the results of these studies [37, 38].

In another study comparing the antibacterial activity of 2% chlorhexidine and different essential oils including *Mentha piperita* against *S. mutans* [39], *Mentha piperita* oil exhibited the highest antimicrobial activity against *S. mutans*, while less activity was found in the control group, chlorhexidine. In contrast to our study, the effectiveness of the antimicrobial agent chlorhexidine was found to be lower in that study, and the difference between the results may be due to methodological differences between the two studies. Vegetable oils and extracts have different contents. The essential oil obtained from the *Mentha piperita* at a rate of 0.1–1% mainly contains menthol (29–48%), menthone (20–31%), menthofuran (6.8%), and menthyl acetate (3–10%) [39]. Therefore, the antimicrobial activity of essential oils and extracts at different levels can be attributed to the main chemical components of the essential oils, which play the main role in antibacterial activities.

According to our study results, the test groups with chlorhexidine added at concentrations of 3%, 5%, and 10% were found to be statistically more effective than all other test groups. Additionally, it was observed that the group containing chlorhexidine at 1% concentration and the group containing *Thymus vulgaris* at 10% concentration exhibited similar levels of efficacy but lower than the groups containing chlorhexidine at 3%, 5%, and 10% concentrations.

In a study by Gonçalves *et al.* [40] investigating the effect of *Thymus vulgaris* essential oil on the growth of *S. mutans*, experimental toothpastes containing 1%, 5%, and 10% concentrations of *Thymus vulgaris* essential oil in ethanol, the same concentrations of *Thymus vulgaris* oil in mineral oil, triclosan (0.25% and 0.5%) in ethanol, and chlorhexidine digluconate (0.06% and 0.12%) in ethanol were tested. As a result of the study, while efficacy was observed at all concentrations of *Thymus vulgaris* essential oil diluted in ethanol, growth inhibition of *S. mutans* was observed only at a 10% concentration of *Thymus vulgaris* essential oil diluted in mineral oil [40]. The difference between that study and our results is thought to be due to the difference in the solvents selected in the study design and the use of essential oil instead of plant extract.

The activities of the plant extracts tested in our study were compared with the negative and positive control groups. According to our results, *Mentha piperita* was found to be effective on *C. albicans* compared to the negative control group but was significantly less effective than fluconazole. Our study results are in line with the results of a previous study [41]. According to our study results, when the MIZ diameters of the concentrations of the tested antimicrobial agents were evaluated against *C. albicans*, the lowest effectiveness was found after the addition of plant extract at 1% and 3% concentrations, regardless of the type. In addition, 5% and 10% concentrations were like each other and showed higher effectiveness than 1% and 3% concentrations.

When the dual interactions of the antimicrobial agents and their concentrations were taken into consideration, it was found that the positive control groups containing fluconazole were significantly effective at all concentrations. No studies comparing these results one-to-one were found in the literature. However, in a study investigating the antimicrobial activity of *Salvia officinalis* and two other plants on *Staphylococcus aureus, Escherichia coli,* and *Candida albicans* using the agar diffusion method [42], it was observed that all concentrations (5, 10, 20, 40%) of all plant extracts used had an inhibitory effect on microbial growth, and zone formation increased as the concentration ratio increased. It is thought that these results are parallel with the results obtained in our study.

In addition to antimicrobial efficacy, our study examined the mechanical properties, including tensile bond strength, tear resistance, and surface roughness of the tissue conditioners with plant extracts added at MIC levels for *C. albicans*. Results showed no significant differences between the control group and the plant extract-containing tissue conditioners. These findings align with studies by Srivastava et al. [43] and Noori and Jaber [44], where the addition of *Origanum oil*, *Neem*, and *Aloe vera* extracts did not significantly affect bond strength in tissue conditioners.

In contrast, studies reporting altered mechanical properties in tissue conditioners containing antimicrobial agents or nanoparticles have reported changes in mechanical properties. Ataol et al. [19] found that the addition of fluorescent carbon nanoparticles from licorice root reduced the tear strength of both acrylic- and silicone-based tissue conditioners, highlighting how nano-additives can modify mechanical characteristics.

In a study investigating the impact of adding *Aloe vera* extract to a heat-cured tissue conditioner on *C. albicans* adhesion and selected mechanical properties, 3% and 10% concentrations were observed to increase tear resistance at the end of 24 hours. However, no statistically significant difference was found between the control and experimental groups [45]. Researchers attributed this result to the potential physical bonding between *Aloe vera* powder and the tissue conditioner powder particles, along with differences in particle size and shape of the *Aloe vera* and tissue conditioner powders.

Higher surface roughness often correlates with increased yeast cell presence [46–48]. This is likely due to the surface acting as a reservoir, where surface irregularities increase microorganism retention, even during denture cleaning, and reduce the likelihood of detachment under shear forces [48]. Additionally, these irregularities may sometimes permit the irreversible adhesion of entrapped microbial cells to a surface [48]. In our study, surface roughness testing revealed that the addition of plant extracts did not affect the surface roughness of the tissue conditioner. A similar study [43] examining the surface roughness of a tissue conditioner with *Origanum oil* addition found a statistically significant difference in roughness between the control and test groups, concluding that *Origanum oil* significantly reduced surface roughness.

Despite using an acrylic-based tissue conditioner in our study, we obtained similar surface roughness results between test groups with plant extract additions and the control group, contrary to the above study. This outcome may be due to the filling of the pores in the tissue conditioner structure by nanoparticles [49] and essential oils [50] used in prior studies. Additionally, the liquid state of the plant extracts in our study might have been ineffective in filling the porous structure of the material since water-based extractions were added to tissue conditioner samples in the current study.

Various instruments, including conventional profilometers, laser-based profilometers, atomic force microscopes, and scanning electron microscopy (SEM), are employed to assess surface roughness in dental materials [51]. Mechanical profilometers have been reported to be adequate for evaluating the surface roughness of restorative materials following polishing [51] SEM is commonly utilized to identify structural irregularities of the dental materials. Several studies have highlighted the significance of employing multiple techniques to comprehensively assess surface characteristics [51, 52]. In this context, in the current study, mechanical profilometer and SEM were used together to evaluate the surface roughness and topography of tested plant incorporated tissue conditioner samples.

This study has certain limitations regarding the evaluation of antimicrobial activity and the effects of these extracts on the mechanical and physical properties of tissue conditioners. In order to identify bioactive constituents and enhance the efficacy of medicinal plant extract combinations, the compounds contributing to the biological activity-whether through synergistic, additive, or antagonistic interactions were not isolated, structurally characterized, and quantified. To minimize the re-isolation of previously identified bioactive compounds, structural screening procedures might be implemented to eliminate such compounds, utilizing high-resolution mass spectrometry (HR-MS), UV spectroscopy, nuclear magnetic resonance (NMR), and tandem mass spectrometry (MS/MS) molecular networking [53].

In addition, increasing the concentrations of plant extracts tested could provide more comprehensive data. Additional comparisons could be made by using different types of tissue conditioners alongside the acrylic-based tissue conditioner used in this study. Only two types of microorganisms were tested, while numerous and diverse microorganisms exist in the oral microflora. Since this study was conducted under *in vitro* conditions, our results may differ from those obtained under *in vivo* conditions in the oral environment. Factors such as the aging process, occlusal forces, and the use of cleaning agents, which affect the material in clinical settings, could not be simulated, potentially leading to variations in study outcomes.

## Conclusion

Within the limitations of this study, the following conclusions were reached:

1. Chlorhexidine digluconate and fluconazole showed the highest antibacterial and antifungal activity *against S. mutans* and *C. albicans*, respectively, compared to all plant extracts, at each corresponding concentration.
2. All extracts exhibited the highest antimicrobial activity at 5% and 10% concentrations.
3. The 5% and 10% *Saliva officinalis* and *Thymus vulgaris* were the most effective concentrations against *C. albicans,* while 10% *Mentha piperita* showed a limited antifungal activity.
4. The 10% concentration of *Thymus vulgaris* is a promising antibacterial agent, as it demonstrated efficacy comparable to that of 1% chlorhexidine digluconate (the positive control) against *S. mutans*.
5. The addition of plant extracts at their minimum inhibitory concentrations (MICs) did not alter the mechanical or physical properties of the tissue conditioners.

## Supplementary Information

Below is the link to the electronic supplementary material.

Supplementary file1 (DOCX 19 KB)

## Data Availability

Data will be made available on request.
