# Supplementary material for: Assessment of mechanical, physical, and antimicrobial properties of tissue conditioners incorporated with plant extracts
Source: Odontology. 2025 Jun 13;114(2):537–52. doi: 10.1007/s10266-025-01132-2 (PMC13053509; doi:10.1007/s10266-025-01132-2)
Supplement: Supplementary file 1 — Supplementary file1 (DOCX 19 KB) [file 10266_2025_1132_MOESM1_ESM.docx]

**Supplementary File.** Tested materials used in the study.

| **Material** | **Trade name** | **Lot #** | **Manufacturer** | **Content and Properties** |
| --- | --- | --- | --- | --- |
| Acrylic-based tissue conditioning and relining material | GC Tissue Conditioner | 2102121 | GC Dental Products  Leuven, Belgium | Powder is composed of polyethylmethacrylate, and the liquid is a mixture of ethyl alcohol, and dibutyl sebacate (plasticizer) |
| Heat-polymerized acrylic resin base material | Lcad Dent Hot Curing Denture Base Material | 20150416 | Hamle Tıbbi Cihazlar Malz. Tic. Ltd.Şti.  İzmir, Turkiye | Powder is composed of methylmethacrylate copolymer, and the liquid is composed of methylmethacrylat, and dimethacrylate |
| Chlorhexidine Digluconate (Antimicrobial agent) | Sigma | BCCD8075 | Sigma-Aldrich, St.Louis, USA | Chlorhexidine digluconate %20 aqueous solution |
| Fluconazole (Antifungal agent) | Sigma | 0000134966 | Sigma-Aldrich Massachusetts, USA | ≥98% (HPLC), powder C₁₃H₁₂F₂N₆O |
| Brain Heart Infusion Broth liquid medium | Condalab | 105122 | Condalab, Spain | Dextrose 2 g/L Disodium phosphate 2.5 g/L  Gelatin peptone 10 g/L  Sodium chloride 5 g/L  Heart infusion 10 g/L  Brain infusion 7.5 g/L |
| Sabouraud Dextrose Broth liquid medium | Millipore | 108339 | Merck, Darmstadt, Germany | %2 dekstrose broth |
